# A Geometric Feature-Based Algorithm for the Virtual Reading of Closed Historical Manuscripts

**DOI:** 10.3390/jimaging9100230

**Published:** 2023-10-20

**Authors:** Rosa Brancaccio, Fauzia Albertin, Marco Seracini, Matteo Bettuzzi, Maria Pia Morigi

**Affiliations:** 1 Department of Physics and Astronomy “Augusto Righi”, University of Bologna, 6/2, Viale Carlo Berti Pichat, 40127 Bologna, Italy; rosa.brancaccio@unibo.it (R.B.); matteo.bettuzzi@unibo.it (M.B.); mariapia.morigi@unibo.it (M.P.M.); 2 National Institute of Nuclear Physics & Istituto Nazionale di Fisica Nucleare, CHNet, Division of Bologna, Via Berti Pichat 6/2, 40127 Bologna, Italy

**Keywords:** ancient handwritten documents, image classification, image segmentation, X-ray tomography

## Abstract

X-ray Computed Tomography (CT), a commonly used technique in a wide variety of research fields, nowadays represents a unique and powerful procedure to discover, reveal and preserve a fundamental part of our patrimony: ancient handwritten documents. For modern and well-preserved ones, traditional document scanning systems are suitable for their correct digitization, and, consequently, for their preservation; however, the digitization of ancient, fragile and damaged manuscripts is still a formidable challenge for conservators. The X-ray tomographic approach has already proven its effectiveness in data acquisition, but the algorithmic steps from tomographic images to real page-by-page extraction and reading are still a difficult undertaking. In this work, we propose a new procedure for the segmentation of single pages from the 3D tomographic data of closed historical manuscripts, based on geometric features and flood fill methods. The achieved results prove the capability of the methodology in segmenting the different pages recorded starting from the whole CT acquired volume.

## 1. Introduction

Digitization of ancient documents is a crucial key step for their preservation and dissemination: the digital copy can be easily studied and shared, minimizing the consultations of the original papers and parchment that will lead to inevitable degradation. Unfortunately, the most ancient and fragile documents cannot be digitized using traditional techniques: the mere opening of these objects is too risky and, sometimes, simply impossible. For these reasons, X-ray Computed Tomography (CT) is emerging as a new approach for digitization. X-ray CT is a well-established methodology for medicine [1,2], industrial applications [3], and, in recent decades, Cultural Heritage studies [4,5,6,7,8,9]. Thanks to the penetration power and the non-invasive nature of X-rays, CT enables the complete imaging of precious objects. Archaeometric analysis can also be carried out with neutrons for imaging or for material characterization [10,11], successfully applied to cultural heritage for various kind of finds, e.g., Egyptian metallic inks on textiles [12], metallic objects, or other archeological items [13].

Reading of ancient manuscripts by X-ray tomography is feasible thanks to the penetration power of X-rays that enables the acquisition of the entire volume of the document, while the chemistry of the most commonly used ancient inks (iron-based [14,15,16]) determines the X-ray contrast for the reading, in a totally non-invasive way. The pioneering research of Mills et al. [17], the impressive ones of Mocella et al. [18] and Burkreeva et al. [19], and the analysis performed in [20,21] demonstrate the feasibility of the technique on ancient and extremely fragile scrolls using synchrotron radiation sources.

However, the limited availability and accessibility of synchrotron facilities compared to normal X-ray tubes motivated a series of feasibility studies using laboratory sources. Examples are the tomographic imaging of Herculaneum papyri [22], of the En Gedi scroll, the oldest scroll in Hebrew outside of the Dead Sea Scrolls [23], as well as of several administrative documents [24], of a book mockup [25], of a soiled bamboo scroll [26], of damaged historical scrolls [27], and of sealed testaments and a handwritten ancient book [28].

The successful outcome of these investigations proved the effectiveness of the technique, also with more traditional and more accessible instruments than a synchrotron facility. In addition, all these studies have shed light on the most challenging step of the technique: the virtual reading of the text, its segmentation, and the extraction of the individual pages from the 3D tomographic volume.

In this direction, several techniques have been proposed, from a fully manual approach [29], to a combination of algorithmic and user-driven operations [30], to a more automatic technique exploiting segmentation, surface modeling and ink projection [31], or the use of a triangular mesh for surfaces characterization [23,32]. More automatic algorithms, such as the topological identification and propagation, were applied by [27,33] on scrolls and, more recently, by [25,26] on book and bamboo scrolls. An extensive study of text segmentation for virtual reading of closed envelopes has been described in [34].

In this work, we propose a new, simple and fast procedure for the segmentation of single pages from the tomographic data of closed historical documents, based on geometric features and flood fill methods.

The aim of the work is to introduce a simple, computationally fast and effective method for the extraction of the pages from a closed book, without any assumptions on their number. Given this premise, we intend to exploit the geometry of a closed book, in which the pages have a specific orientation: they are ideally planar and parallel each other. For this reason, the curved parts of the folded document are excluded from the analysis. This last problem has been investigated by, e.g., [34]. First, to test the effectiveness of our method, we simulated a mock up folding a single page Italian handwritten document dating to 1679. In addition, we applied our segmentation algorithm to a sealed Venetian testament [29].

Thanks to its short computation time (in the order of some minutes, depending on the size of the data), our proposed method allows the user to verify and set the best combination of parameters for each specific case. They have an immediate interpretation, such that the operator can easily vary them, figuring out which the best choice can be. This aspect is particular important, because it is not possible to state a priori which is the optimal combination, as evident from other state-of-the-art works on the same topic (see, e.g., [33,34]). Furthermore, the execution time can be additionally reduced, being the written software adaptable to multiple processors (parallel calculus).

In addition, the new semi-automatic algorithm is interactive because it allows us to preview the final classification.

## 2. Document Sample and Tomography

### 2.1. Document Folding for Book Simulation

**Figure 1:**
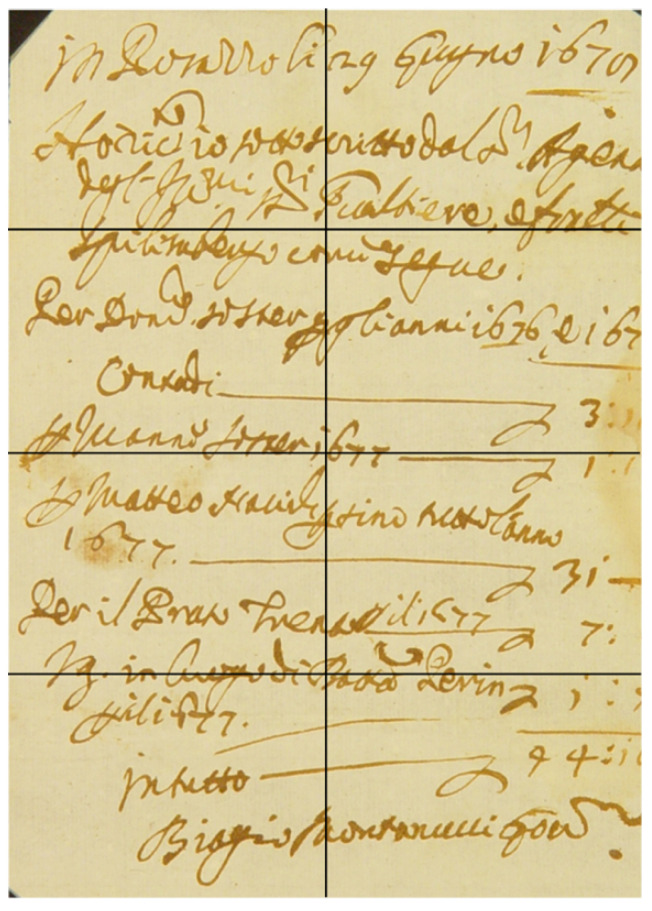
Picture of the unfolded 1679 manuscript. The black lines show the trace lines used to fold it in order to create the “eight pages”.

To realistically simulate the case of a closed book, a private Italian handwritten document, shown in Figure 1, dating to 1679 and 21×15 cm^2^ in size has been used. The manuscript support is made of paper and the ink is iron-based. Thanks to the heavy elements present in this common European ancient ink type (i.e., iron), the writings have a high X-ray contrast, allowing good imaging of the text. To simulate a multi-page document, the manuscript was folded three times, along the straight lines marked in black in Figure 1, achieving a simulated “eight pages book”, that was put inside a plastic envelope and tied with a string to protect it and keep the fold stable.

The choice to perform the analysis on this kind of folded handwritten documents is justified by the fact that, in historical archives, many manuscripts present similar folding and morphology, such as ancient last wills.

The CT analysis was carried out at Ecole Polytechnique Fédérale de Lausanne—EPFL (Switzerland) using a lab-based micro-CT system with the tomographic settings shown in Table 1.

### 2.2. The Tomographic Reconstruction

**Figure 2:**
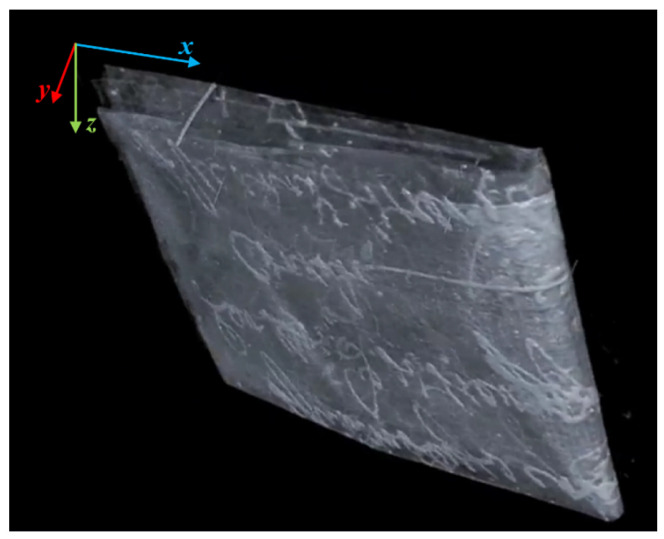
X-ray 3D tomographic reconstruction of the full folded document. The writings are clearly visible, but the page superimposition makes the document unreadable without ad hoc processing.

The tomographic scan resulted in a volume of 3586×2740×178 voxels (respectively x,z,y of Figure 2), with voxel size of 15 μm. The reconstruction was performed with the Feldkamp algorithm (FDK). A 3D rendering of the manuscript is shown in Figure 2 (the tomographic reconstruction and analysis for the segmentation were performed using PARREC [35], a software internally developed at the University of Bologna).

The computer used for the reconstructions and the elaborations of the data is an assembled computer with Windows 10 Pro x64, CPU Intel(R) Core(TM) i7-8700k @3.70 GHz, RAM 16 GB. The following execution times will refer to the system working without any parallelization.

### 2.3. The Page Segmentation Problem

**Figure 3:**
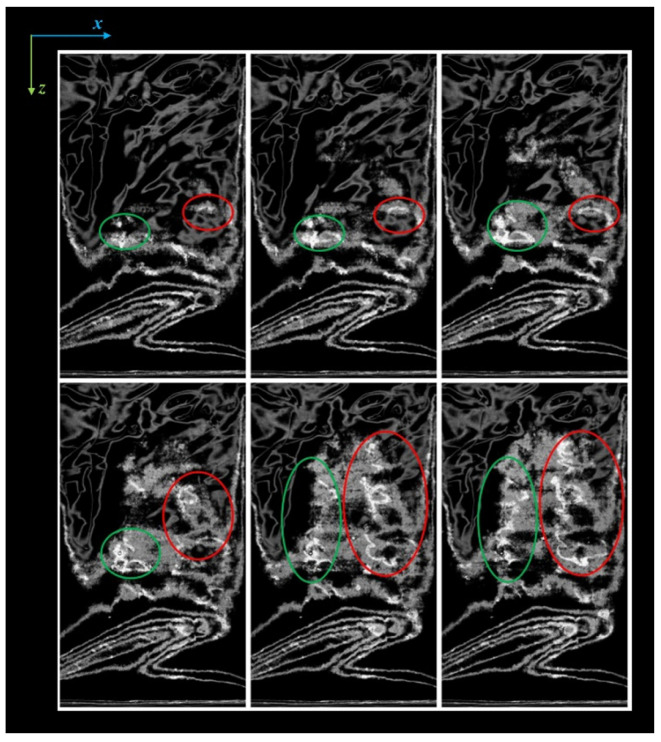
Sequence of six consecutive slices. Two text lines are highlighted by green and red circles, showing their partial reading along different slices.

Although the writings are clearly visible in the volume rendering, the non-planarity of the pages makes the reading impossible by simply navigating inside the tomographic volume. An example of this effect is shown in Figure 3. Here, each slice/plane contains only a small portion of the text—and most often of a single letter. This effect becomes worse in case of a poor conservation state or more ancient documents, where the pages are often bent, slightly curved, and include roughness and creases. In addition, the higher the tomographic resolution, the more difficulties caused by every little roughness of the page paper.

**Figure 4:**
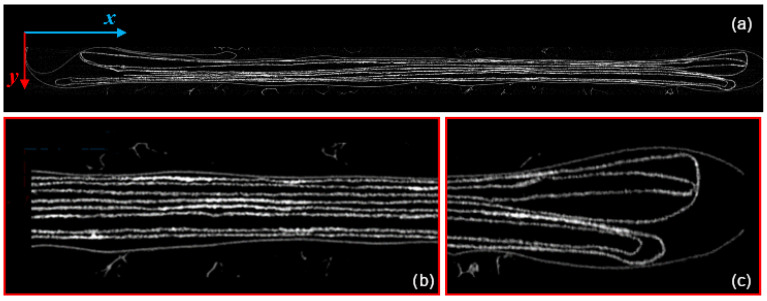
CT reconstruction of the document on the x/y plane. (**a**) The whole slice. (**b**) Zoom of the central area of the slices where the brightest pixels correspond to the ink (writings), the light gray-levels to the pages and the darker ones to the background. (**c**) Detail of the folded area.

The preliminary step is to identify the different pages, a target almost impossible to achieve from the point of view shown in Figure 3. The easiest way is to section the tomographic volume along another direction, choosing a plane that is perpendicular to the pages, as shown in Figure 4.

## 3. Algorithm

**Figure 5:**
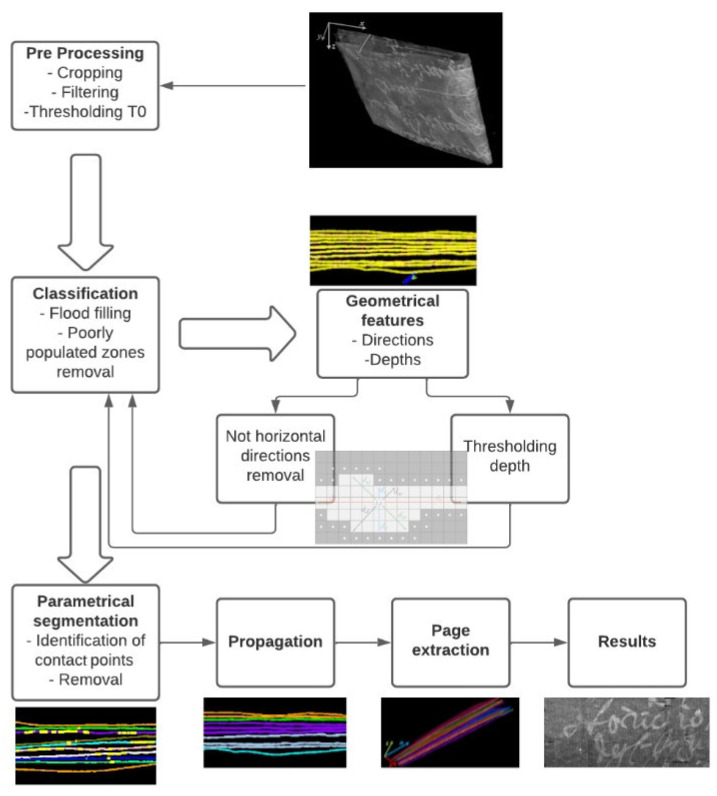
Flowchart of the whole procedure.

By analyzing the tomographic images on the x-y plane shown in Figure 4, it is possible to identify two main geometric characteristics: the pages are horizontally disposed (along *x*-axis) and have a small thickness if compared with their horizontal length. Then, the basic idea driving our approach is to extract the pages through their main direction (*x*-axis) and thickness (*y*-axis). As illustrated by the flowchart in Figure 5, the proposed method can be divided into six steps:

(1) Pre-processing;
(2) Classification;
(3) Geometric characteristics definition;
(4) Parametric segmentation and iteration;
(5) Propagation;
(6) Page extraction.

The pre-processing step aims to separate the regions belonging to the pages from the background (details are provided in Section 3.1).

Once all the pixels of the image have been filtered, the classification step follows (see Section 3.2). The image is analyzed in order to assign to contiguous zones a progressive number that defines the specific page each pixel belongs to. If the pages are not connected along the *y*-direction, this step is easy to perform. Unfortunately, at this point, even connections of a few pixels can bring to page misclassification. For a correct procedure, it is therefore necessary to consider the geometric characteristics of each region. In this sense, the direction, numerosity, thickness, and other geometric features are defined and calculated. The contact points are represented by pixels placed in thicker zones than the average. The segmentation is then performed based on a set of parameters exploiting the geometric characteristics previously calculated. This step is iterated up to the complete segmentation of the image pages—eight in this case (plus two envelopes). All the described operations are initially performed on a single x–y slice chosen around the center of the object, as in the example provided in Figure 4b, around the center of the object. The result achieved for a single image is then propagated along the *z* direction, covering the entire CT volume.

### 3.1. Pre-Processing

**Figure 6:**
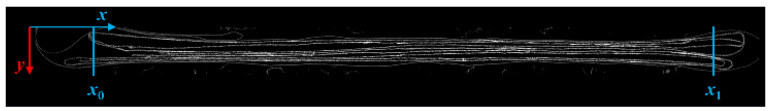
A cropped slice: at the ends of the image, the two blue lines indicate the limits of cropped areas. For each section, only the parts contained between $x_{0}$ and $x_{1}$ will be used in the subsequent elaborations.

Before identifying and extracting the pages of the manuscript, it is necessary to reduce the background noise and prepare the data for the subsequent elaboration. To simplify the problem, the first step of the pre-processing stage is the cropping of the images. Differently from [34], the folding of the sheets is excluded from the analysis and it remains located at the extremes of the image, as shown in Figure 6.

Our goal is to provide a methodology that gives better results in terms of contrast of the final reconstructions than [34] while reducing the computational cost.

In fact, considering that a closed book is ideally characterized by a planar geometry of the pages, one of the aims of the cropping procedure is to reduce the amount of data to be elaborated in favor of a lower execution time. We will denote the cropped images by $C_{z}\left(x,y\right)$.

**Figure 7:**
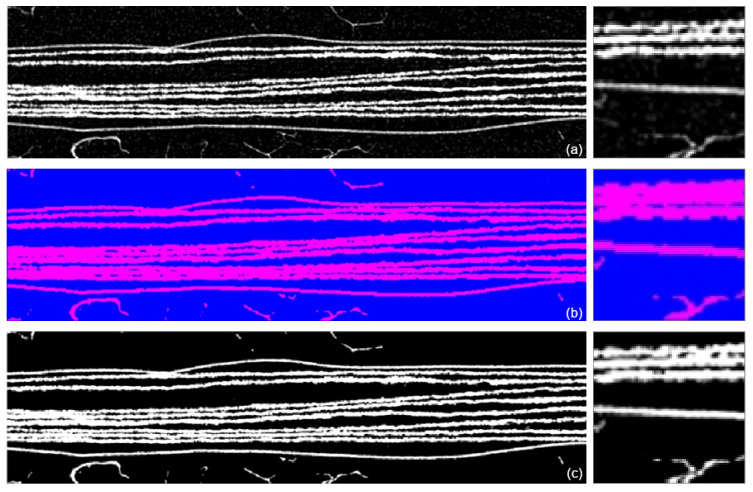
Example of pre-processing (details in the square images on the right column). (**a**) The original slice with normalized gray levels to highlight the background noise. (**b**) The classification after the two filters application: in purple the pixels that assume values over the threshold while in blue the background pixels. (**c**) The same slice after the pre-preprocessing operated in (**b**), with normalized gray levels (eight pages and two envelopes).

After the image cropping, a noise filter is applied to reduce spurious components: the result is shown in Figure 7a. To identify noisy pixels, we used a segmentation approach already successfully applied in [36], but modified by different filters. The method consists of five steps:

(a) Filtering $C_{z}\left(x,y\right)$ to achieve a filtered image $F_{z}\left(x,y\right)$;
(b) Calculating the histogram of $F_{z}\left(x,y\right)$;
(c) Choosing a “pruning” threshold $T_{0}$ on the histogram to separate the useful signal (i.e., the pages) from the background;
(d) Using $T_{0}$ to decide which pixels to keep and which to discard from the original image.

Filtering is a well-known image processing method [37,38], whose results depend on the filter characteristics. The filters chosen in our filtering stage, with the aim to improve the quality of the segmentation of the pages, are the standard Maximum and Median ones, opportunely modified for our particular scopes. We define the S(x,y)=[x−Δx,x+Δx]×[y−Δy,y+Δy] with Δx, Δy=1 as the subset that individuates the square neighborhood of a given point of coordinates (x,y), characterizing the used filters. At the first step, the maximum filter results in:

(1) $$G_{z}\left(x,y\right)=max_{\left(i,j)\in S(x,y\right)}C_{z}\left(i,j\right)\;,$$

where $C_{z}$ are the cropped images (see Figure 6). Initializing a matrix $F_{z}\left(x,y\right)$ of the same size of $C_{z}\left(x,y\right)$ with all zero values, each initial value is increased by one for each pixel of the neighborhood that assumes a value equal to $G_{z}\left(x,y\right)$. This process is iterated for each pixel of $C_{z}\left(x,y\right)$. Doing so, at the end of the procedure, the pixels that have repeatedly assumed the maximum value in the neighborhood *S* will have the highest values. At the end of the whole scanning process, to preserve the signal belonging to the pages and, at the same time, to reduce the noise, all pixels where $F_{z}\left(x,y\right)>0$ have been marked as 1 in a binary matrix and a pointwise logic AND has been used, achieving $C_{1}\left(x,y\right)$.

In this way, $C_{1}\left(x,y\right)$ becomes a binary mask in which the “white” points discriminate the pages from the background, while $C_{z}\left(x,y\right)$ contains the number of points in the neighborhood having their value equal to $G_{z}\left(x,y\right)$.

Then, the same procedure described in the previous points (a) to (d) is applied using a Median filter, calculating the median in the neighborhood *S*. At the end, a segmentation threshold $T_{0}$ is chosen to suppress the lower values pixels in the background. The described filtering procedure has been applied to our specific problem choosing for both filters Δx, Δy=1 and a $T_{0}$ of around 6% of the maximum gray value. An example proving the effectiveness of the proposed pre-filtering procedure is shown in Figure 7 (values are normalized to improve the visual readability). This algorithm decreases the noise without damaging the page signal at higher values.

### 3.2. Classification

The classification algorithm exploits the definition of four- and eight-connections; a pixel is four-connected to an adjacent pixel if it shares at least one side with it; a pixel is eight-connected to an adjacent one if it shares one side or a corner with it (see [37]).

After the pre-processing step, all the pixels have been marked as signal or background. Operatively, the algorithm starts to scan the image pixelwise and test if the current pixel belongs to a page: if not, the scan continues until a page pixel is found. This first pixel is marked as belonging to zone 1. Then, the procedure groups together the pixels belonging to the same zone, according to the 8-connectivity definition. The process is iterated until the complete classification of each pixel is achieved, to individuate multiple distinct connected zones (i.e., the different pages). This procedure is commonly known as flood fill algorithm [39].

At the end of the scanning, a number $N_{0}$ of zones will have been found (in our application, it results that $N_{0}>>10$, being eight the effective known number of pages and two the number of the envelopes). This is due to many small areas wrongly classified as page zones, caused by artifacts or small impurities.

**Figure 8:**
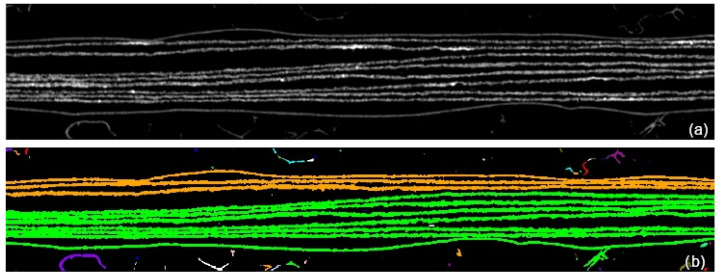
Result of the classification step for a zoomed area of one slice $I_{z}\left(x,y\right)$. (**a**) The background is in black, while the gray pixels belong to the pages. (**b**) This image is in false colors: different colors indicate different classified zones. The pages are classified into two zones only (in orange and green). The contact points between the pages wrongly causes the grouping of multiple zones in a single one. Other colors highlight impurities and noise that need to be eliminated: these small areas causes $N_{0}$ to be much higher than the effective number of pages (8 in our case, plus 2 envelopes).

After the suppression of the spurious areas, characterized by a reduced extension, the number of connected zones results lower than the number of pages. An example of page classification is shown in Figure 8b, and achieved processing in Figure 8a. If the pages were completely separated, there would be no need to continue processing, and the software could go directly to the data extraction step, but, as shown in Figure 8, they are in contact in some points and the algorithm tends to underestimate the number of actual pages (only two instead of eight). This is due to the compression of the manuscript and the lack of space between the pages themselves. Further steps of the algorithm are needed for the identification and removal of contact points and impurities.

### 3.3. Geometric Characterization

**Figure 9:**
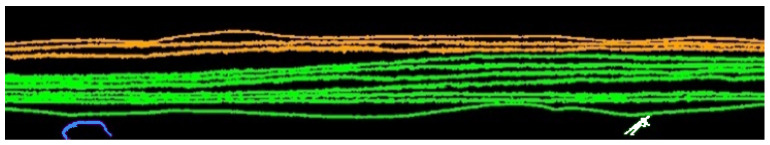
Result of the classification after poorly populated zones removal: there are now four classified zones $\left(N_{1}=4\right)$, i.e., two pages areas (in green and orange) and two spurious areas (in blue and white).

The area of each zone is the first geometric feature to be studied. In fact, we expect that the poorly extended zones belong to noise or impurities, while the ones relative to the pages must have a very large area, especially considering that multiple pages can be in contact and are, then, grouped together. In this preliminary step, the classified zones with area less than 0.01% of the total number of pixels in the image are suppressed, assigning them to the background. An example of this stage is shown in Figure 9. At the end of the poorly populated zones suppression, the new number of remaining ones reduces to $N_{1}<N_{0}$ (in our particular case, $N_{1}=4$).

In this procedural phase, in order to identify contact pixels, geometric features will be defined and calculated. For each signal pixel $\left(x,y\right)\in G_{z}\left(x,y\right)$, its distance from the closest background pixels can be calculated in eight directions: up, down, right, left and the four diagonals. These distances are defined using the minimum of the well-known Euclidean metric, in the following way:

(2) $$d_{\theta }\left(\left(x,y\right),\left(x_{b\theta },y_{b\theta }\right)\right)=min_{\theta }\left\{{\left({\left(x-x_{b\theta }\right)}^{2}+{\left(y-y_{b\theta }\right)}^{2}\right)}^{\frac{1}{2}}\right\}>0$$

with

$$\theta \in \{0,\frac{\pi }{4},\frac{\pi }{2},\frac{3}{4}\pi ,\pi ,\frac{5}{4}\pi ,\frac{3}{2}\pi ,\frac{7}{4}\pi \}.$$

**Figure 10:**
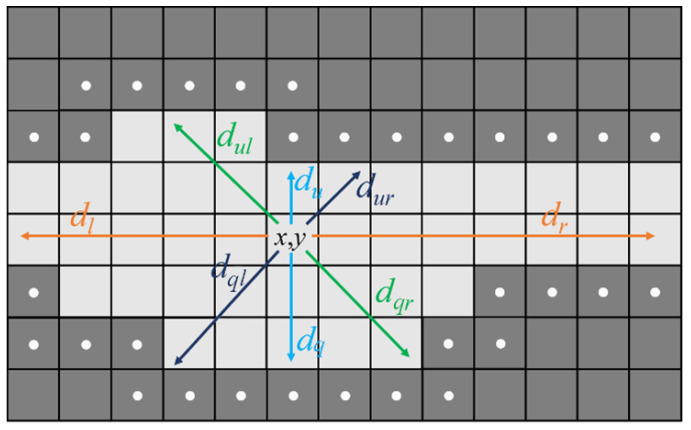
Example of calculation of depths in all eight directions (orange shows the horizontal direction, cyan the vertical, black the positive diagonal and light green the negative diagonal). In this case, the highest depth value is in the horizontal direction, while the lowest one is in the vertical (cyan) direction: the point at coordinates P(x,y) is then assigned to a horizontal zone and with a depth equal to the vertical one. The set Φ of the background pixels closest to signal zone are indicated by a light gray point.

Referring to Figure 10, where pedexes are introduced to simplify the notation, it is possible to define:

(3) $$depth_{hor(x,y)}:=d_{l}\left(x,y\right)+d_{r}\left(x,y\right)$$

(4) $$depth_{ver(x,y)}:=d_{u}\left(x,y\right)+d_{q}\left(x,y\right)$$

(5) $$depth_{posdiag(x,y)}:=d_{ur}\left(x,y\right)+d_{ql}\left(x,y\right)$$

(6) $$depth_{negdiag(x,y)}:=d_{ul}\left(x,y\right)+d_{qr}\left(x,y\right)$$

where *hor*, *ver*, *posdiag*, *negdiag*, respectively, stay for horizontal, vertical, positive diagonal $\left(\theta \in \left\{\frac{\pi }{4},\frac{5}{4}\pi \right\}\right)$, negative diagonal $\left(\theta \in \frac{3}{4}\pi ,\frac{7}{4}\pi \right)$. In particular:

(7) $$d_{r}\left(x,y\right)=d_{\left(\theta =0\right)}\left(x,y\right)$$

(8) $$d_{ur}\left(x,y\right)=d_{\left(\theta =\frac{\pi }{4}\right)}\left(x,y\right)$$

(9) $$d_{u}\left(x,y\right)=d_{\left(\theta =\frac{\pi }{2}\right)}\left(x,y\right)$$

(10) $$d_{ul}\left(x,y\right)=d_{\left(\theta =\frac{3}{4}\pi \right)}\left(x,y\right)$$

(11) $$d_{l}\left(x,y\right)=d_{\left(\theta =\pi \right)}\left(x,y\right)$$

(12) $$d_{ql}\left(x,y\right)=d_{\left(\theta =\frac{5}{4}\pi \right)}\left(x,y\right)$$

(13) $$d_{q}\left(x,y\right)=d_{\left(\theta =\frac{3}{2}\pi \right)}\left(x,y\right)$$

(14) $$d_{qr}\left(x,y\right)=d_{\left(\theta =\frac{7}{4}\pi \right)}\left(x,y\right)$$

In that way, each pixel belonging to a zone is characterized by four geometric features, i.e., its distances (depth) from the background pixels in the four main directions (horizontal, vertical, positive and negative diagonal).

It is possible now to recognize to which page each extracted point belongs, introducing the PrincipalDirection and the PrincipalDepth as:

(15) $$\begin{array}{c} PrincipalDirection\left(x,y\right)=arg\max_{k}depth_{k} \end{array}$$

(16) $$\begin{array}{c} PrincipalDepth\left(x,y\right)=arg\min_{k}depth_{k} \end{array}$$

with k∈{hor,ver,posdiag,negdag}.

To visualize the concept behind the geometric features definitions, see Figure 10.

**Figure 11:**
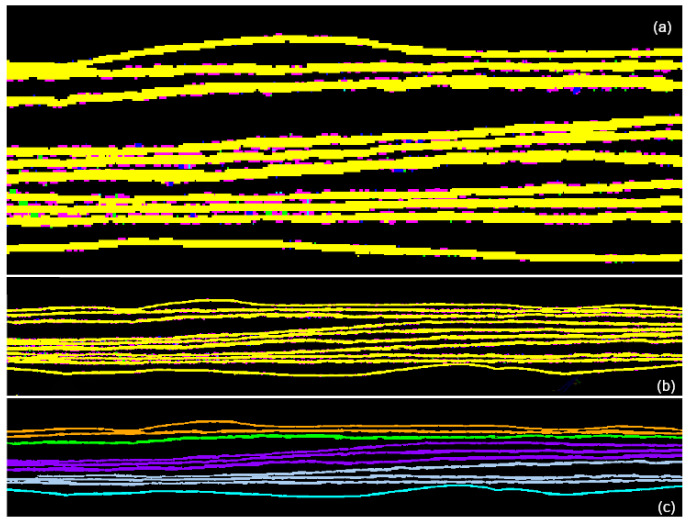
(**a**) False-colored visualization of the geometric features computed for each pixel of Figure 8: the yellow color individuates the pixels for which the Principal Direction is horizontal, cyan the ones for which the Principal Direction is vertical, blue the ones for which the Principal Direction is positive diagonal and, finally, light green the ones for which the Principal Direction is negative diagonal. Fuchsia pixels are instead pixel with Principal Direction different from their neighbors and, consequently, they are most likely contact pixels. (**b**) The same as (**a**) for the whole area of Figure 8. (**c**) Result of the classification after the elimination of the individuated contact pixels: five different zones are clearly distinguishable in different false colors, individuating distinct pages (orange, green, purple, light gray and cyan).

Geometric features are particularly useful at the first step of the separation process. Applying the calculation of the directions and depths to all the pixels in the image, it is possible to analyze their geometric characteristics. The pixels belonging to the pages have a predominant horizontal directionality. Contact points can be identified in the first instance as those pixels with different Principal Direction as compared to their neighbors (see the fuchsia pixels in the zoomed area in Figure 11a). Switching the association of all the contact points from signal to background and applying again the flood fill algorithm, it is possible to segment more appropriately the zones belonging to different pages. At the end of the procedure, the number of separated pages is $N_{2}>N_{1}$.

Despite the operated filtering, points of contact between the pages still persist, such that refining the procedure is necessary to identify and eliminate them. This further operation is illustrated in the next section.

### 3.4. Parametric Segmentation and Iteration

**Figure 12:**
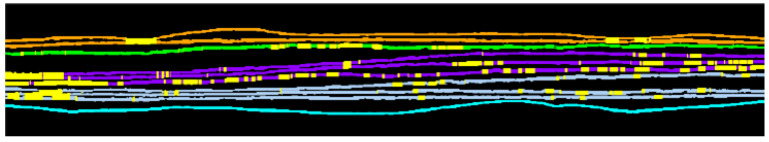
Example of the identification of probably contact pixels, found based on mean depth analysis: they are highlighted in yellow, while pages are highlighted in false colors—the same as in Figure 11c.

Generally, at the end of the previous step, we do not yet have a correct separation of all the manuscript pages. In fact, several contact points are present at the end of the previous step (see Figure 12). These contact points represent a challenging problem for the correct identification of the single pages. For this reason, parametric segmentation is needed using the pages’ depth. Exploiting Equation (1), we can calculate the mean depth values relative to each classified zone. The results are listed in Table 2.

At this point, the pages can be separated introducing another threshold $T_{1}$: contact points are pixels with depth greater than the average depth plus the standard deviation. We can choose a threshold $T_{1}$ that is a function of the average of $Md_{j}$:

(17) $$T_{1}=k\frac{\sum_{j=1}^{N_{2}}Md_{j}}{N_{2}}$$

where $Md_{j}$ is the mean depth for each zone individuated by the $N_{2}$ number of zones, and *k* is a constant. Experimentally, a suitable value for *k* is equal to 1.3. In this way, the pixels of a thickness greater than 1.3 $T_{1}$ are identified in the first instance as pixels that could be of contact. The calculated mean is (3.6±0.5) pixels. An example of the candidate contact points can be observed in yellow in Figure 12.

**Figure 13:**
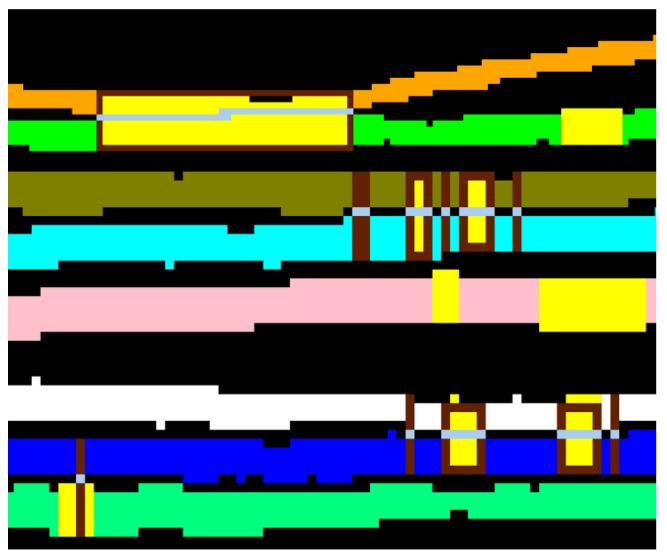
Example of how the separation algorithm works: the yellow areas are the set of pixels identified as dubious contact, the brown bordered rectangles are the edges of the areas classified as effectively contact areas to be separated. The separation algorithm analyzes the number of edge pixels to the right and left of the squares and connects them with a straight line. Light gray pixels immersed in the brown rectangles (e.g., between the third an fourth pages) are contact points identified with the separation method.

**Figure 14:**
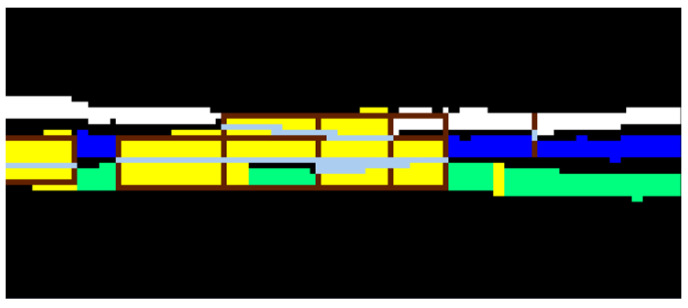
Example of zoomed contact areas of three pages: the pages are white, blue and green (see the right side). The dubious points are highlighted in yellow, the brown rectangles are superimposed and the separation lines are in light gray.

Looking at Figure 12, we can see how all the contact pixels are correctly identified, even if the algorithm classifies also as dubious all the pixels belonging to a thicker area, so that we need to separate effective contact points areas from naturally thicker zones of the pages. The method ideated to distinguish the two cases is based on the identification of the dubious rectangular areas and on the number of the boundary points: if there are points in both left and right sides that border the background (except the four vertices), the identified area is a contact area, otherwise it is classified as a naturally thicker area in the page. The method can be summarized as follows:

(1) Scanning the image for the first dubious point (belonging to yellow areas in Figure 12);
(2) Searching for dubious points close to it to define a surrounding dubious area (yellow areas in Figure 12 and Figure 13);
(3) Identifying the four vertices (minimum and maximum couples of coordinates) of the dubious area: these values define a rectangle;
(4) If there is at least one pixel contiguous to the background both on the left and the right side of the ambiguous zone, the area is classified as contact area (brown bordered rectangles in Figure 13 and Figure 14), otherwise it is not modified. It should be noted that if there are two neighboring pixels in the two sides, but one or both are in the vertices of the rectangles, the area is correctly ignored (see, for example, yellow areas without brown borders in the pink page of Figure 13).

**Figure 15:**
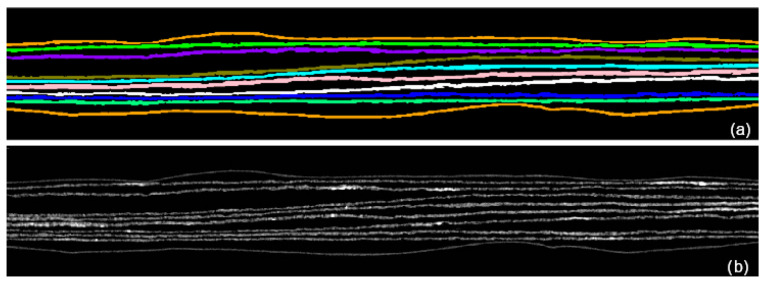
The segmentation of all the pages, correctly separated. (**a**) The segmentation mask in which each page is highlighted with a different color. (**b**) The original data segmented by means of the segmentation mask shown in (**a**): all the pages have been correctly separated from the background.

The method just illustrated starts by analyzing the first dubious point found in the image and continues (looking for a rectangle with the characteristics of step 4) for each point in the image. We remark that, regardless of whether dubious areas are found or not, the method continues to search for contact zones without discarding the points already analyzed. This approach is very important, because it allows us to analyze overlapping rectangles that could otherwise be lost during the analysis. An example of overlapping rectangles can be seen in Figure 13, in the contact area on the left of the last two pages (blue and green ones). Here, there are two dubious areas: one is located only on the green page (in yellow), the other is crossing the two pages (in brown). The first one is discharged, the second one is processed. This good result could not be reached if the dubious points already analyzed were skipped during the processing. Once the contact areas have been identified, they have to be corrected. It is relatively simple to separate the areas that have only one pixel bordering the background on both the left and the right sides (there are only two pages touching each other). An example of this case is in Figure 13, in the orange and green pages (first and second from top, respectively). On the other hand, the situation in which three or more pages touch each other is more complicated. An example of this case is illustrated in Figure 14, in which there are three pages touching each other, with three contact areas. To obtain a correct separation, it is necessary to couple the border points for each corresponding layer. Then, the found pixels are to be connected in order from top to bottom and they are classified as background. At the end of this step, performing the classification again, all the pages are correctly separated. The result is shown in Figure 15. A summary of all the geometric features is reported in Table 3: all the internal pages have a similar thickness, while the first and the last ones are thinner, being pages of the external envelope. A segmentation mask, relative to a single cross-section of the CT data, is the result of the classification procedure.

### 3.5. Propagation

At the end of the previous step, all the ten pages have been separated, but only in one slice (x-y plane). To complete the procedure, the segmentation must be propagated to all the tomographic slices of the document. The total number of slices in our case is nS=3586, while the selected slice to start the segmentation procedure described above is the 2504th. Exploiting the classification described in the previous step, we compute a mask $M_{k}\left(x,y\right)$ defined as follows:

(18) $$M_{\left(k+1\right)}\left(x,y\right)=\left\{\begin{array}{c} 0\;\;if\;\;I(x,y)\in background\\ p\;\;if\;\;I(x,y)\in page\;p \end{array}\right.$$

with $p=\left\{1,2\ldots ,10\right\},0\leq x<W^{\prime },0\leq y<H,0\leq k\leq nS$ and I(x,y) denoting the pixel value of coordinates (x,y). With the word *background* we denote every point not classified as pages in the previous step.

Since the tomographic analysis was performed at high spatial resolution (voxel size of 15 μm), making the same assumption of [33], we expect that page pixels, individuated in one slice, will have small displacements when passing to the bordering ones, such that their position will not have a considerable variation during the propagation step. Propagating the segmentation to the other slices is convenient because, assuming it has an initial page correctly segmented, this would reduce the probability of misclassification. In fact, the propagation algorithm bases the extraction on the previously segmented pages. For this reason, instead of processing each page per se, a propagating approach is preferable, because it is more stable and less sensitive to spurious variations among subsequent slices.

Assuming that, the segmentation mask defined for a certain slice can then be propagated to the whole document with small adjustment slice by slice. We compute all geometric features in the bordering slice with (19), such that only the page pixels close to the background, at coordinates (x,y), having $d_{\theta }\left(x,y\right)\leq 2$, will be analyzed in the next slice. First, the k+1 (or k−1, depending on the direction chosen for the propagation) slice is loaded into memory; then, the mask obtained for the previous slice $\left(M_{k}\right)$ is assigned to each pixel, and all pixels that are away from the background are analyzed. If the pixel in this new slice has a value below the classification threshold $T_{0}$ (the same threshold used in the pre-processing step A), it is classified as a background pixel in $M_{k}+1$; if the pixel is classified as background in $M_{k}$, but has a value greater than $T_{0}$ in the k+1 slice, it is considered as belonging to the current page. This operation is carried out for all the pages, and it concludes by saving the new mask $M_{k}+1$. Then, the procedure is iterated for the other slices along the entire reconstructed sequence. The computation of the mask for the k+1 slice can be formalized as follows:

(19) $$M_{\left(k+1\right)}\left(x,y\right)=\left\{\begin{array}{c} M_{k}\left(x,y\right)\;if\;I_{k}\left(x,y\right)\geq T_{0}\wedge I_{\left(k+1\right)}\left(x,y\right)\geq T_{0}\;,\\ M_{k}\left(x,y\right)\;if\;I_{k}\left(x,y\right)<T_{0}\wedge I_{\left(k+1\right)}\left(x,y\right)<T_{0}\;,\\ p\;if\;I_{k}\left(x,y\right)<T_{0}\wedge I_{\left(k+1\right)}\left(x,y\right)\geq T_{0}\;,\\ 0\;if\;I_{k}\left(x,y\right)\geq T_{0}\wedge I_{\left(k+1\right)}\left(x,y\right)<T_{0}\;, \end{array}\right.$$

with $p\in \left\{1,2\ldots ,10\right\},0\leq x<W^{\prime },\;\;0\leq y<H,\;\;0\leq k\leq nS$.

Note that this step of the algorithm can also segment pages that are close to each other and not separated by the background.

**Figure 16:**
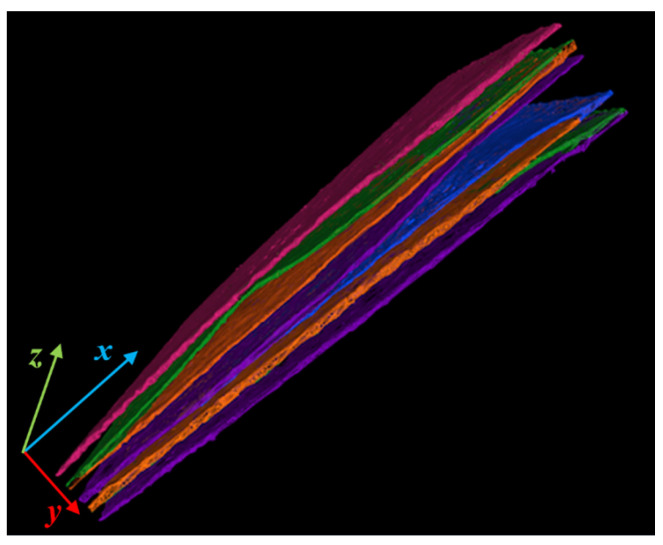
Visual example of the expected results of classification mask applied to the whole volume.

In Figure 16, the 3D result of the generation of the segmentation mask after the propagation is presented.

### 3.6. Page Extraction

Once the segmentation mask has been propagated to the whole dataset, the last step consists of the page extraction. For each slice in the sequence, the classification of each pixel is calculated in the previous step in $M_{k}\left(x,y\right)$. Each page $P_{p}\left(x,k\right)$ is then computed, reading the whole sequence as follows:

(20) $$P_{p}\left(x,k\right)=\left\{\begin{array}{c} \frac{\sum_{y_{1}\left(p,k\right)}^{y_{2}\left(p,k\right)}I_{k}\left(x,y\right)}{y_{2}\left(p,k\right)-y_{1}\left(p,k\right)+1}\;if\;M_{k}\left(x,y\right)=p\;,\\ 0\;\;otherwise \end{array}\right.$$

where $y_{1}\left(p,k\right)$ is the first pixel in the image $I_{k}$ along the *y*-axis (starting from 0 to height-1 (H)) at fixed *x*, for which $M_{k}\left(x,y\right)=p$ and $y_{2}\left(p,k\right)$ is the latest one, and 0≤x<3586, 0≤p≤10, 0≤k<2334.

In other words, when $M_{k}\left(x,y\right)=p$, the value of $P_{p}\left(x,k\right)$ is the mean through the thickness of the pages.

It is possible for some values of *k* that no pixel has $M_{k}=p$, and then it is very important to set the relative values to zero to preserve spatial disposition along the sequence in the page extraction.

We highlight that the binary mask $M_{k}$, computed in the previous step, is only used here to retrieve the pixels from the CT original data, being applied as a sort of pointwise AND operator.

**Figure 17:**
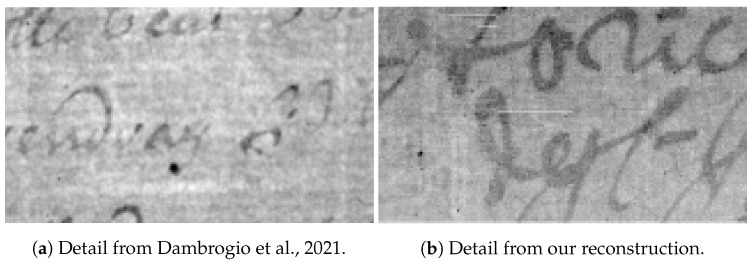
Comparison between two details: (**a**) coming from Dambrogio et al., 2021 [34]; (**b**) coming from our reconstruction.

A qualitative comparison of our reconstructions with a sample image taken from [34] is provided in Figure 17.

## 4. Results

**Figure 18:**
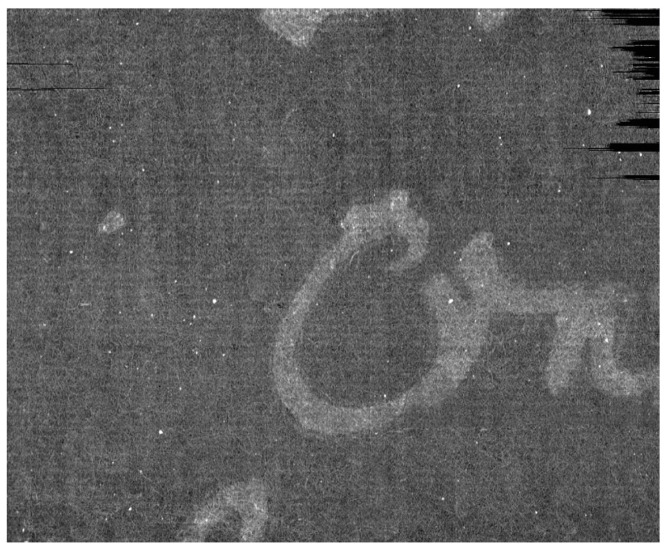
Detail of a letter. The texture of the paper can be seen very well, as well as the contrast with the ink. The small bright spots are ink and paper inhomogeneities. Note how the texture of the document has also been correctly reconstructed.

**Figure 19:**
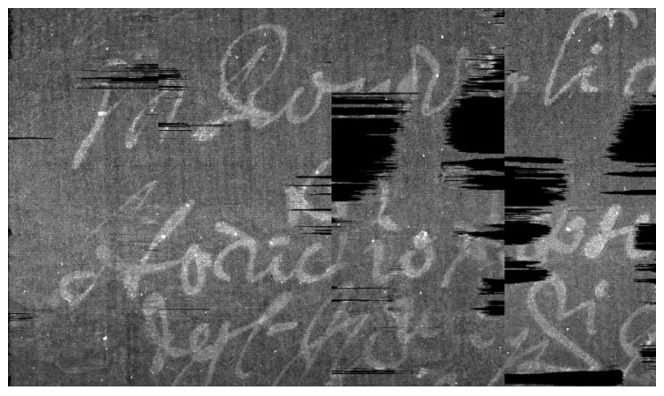
Page 1 extracted after the elaboration of the tomographic reconstruction. The black zones represent areas that the algorithm has mistakenly recognized as background.

**Figure 20:**
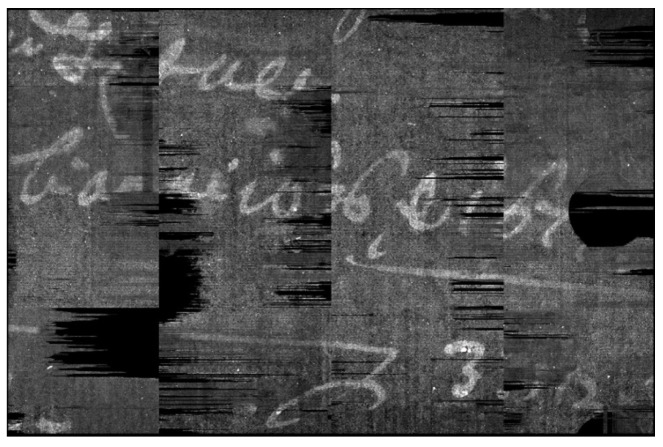
Page 2 extracted after the elaboration of the tomographic reconstruction. The black areas are due to the failure of the page propagation step.

Two extracted pages are shown in Figure 18 and Figure 19. The black areas in Figure 19 and Figure 20 are due to the failure of the propagation step (e.g., in those pixels with low value), while the horizontal black area in Figure 18 is due to the crop in proximity of the folds, where the geometry considerably changes. Moreover, attenuation in the recorded signal can cause a wrong classification of some pixels: if this behavior propagates, then some misclassified areas can appear, like, e.g., the vertical black ones in Figure 19. In our analysis, we are interested in extracting the planar zones of the text. In case of CT of whole books, this problem is avoided thanks to the constructive morphology of the book itself, where the pages are planar to certain extent and where no text appears on the binding of the pages. On the contrary, in books analysis, we expect less air space between pages; therefore, it will be necessary to test and adapt the method to books in future.

**Figure 21:**
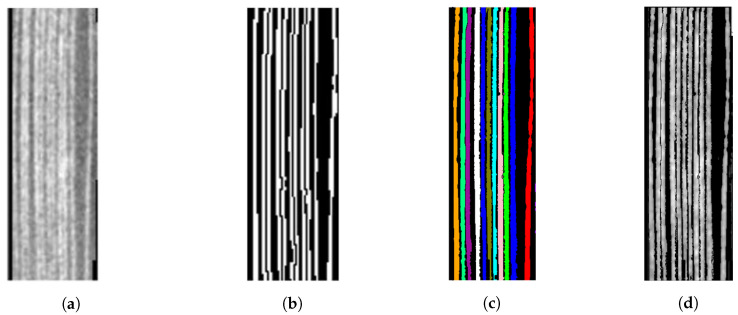
Comparison of our method with the results achieved in [26]. (**a**) The initial 3D reconstruction of the closed manuscript; (**b**) the final segmentation achieved in [25]; (**c**) the segmentation achieved by our method: each color corresponds to a different page; (**d**) the profiles of the pages segmented by our method.

In Figure 21, the comparison between the result achieved by the segmentation algorithm proposed in [25] and our method is provided. Both the algorithms segment 11 different final areas, and it is possible to observe a good regularity of the pages profile extracted by our method (b) to compare it with what achieved in [25] (d).

Moreover, our method is computationally fast and employs only 1.5 s for the segmentation of a single slice, with a total time for the extraction of 5 min and 27 s.

**Figure 22:**
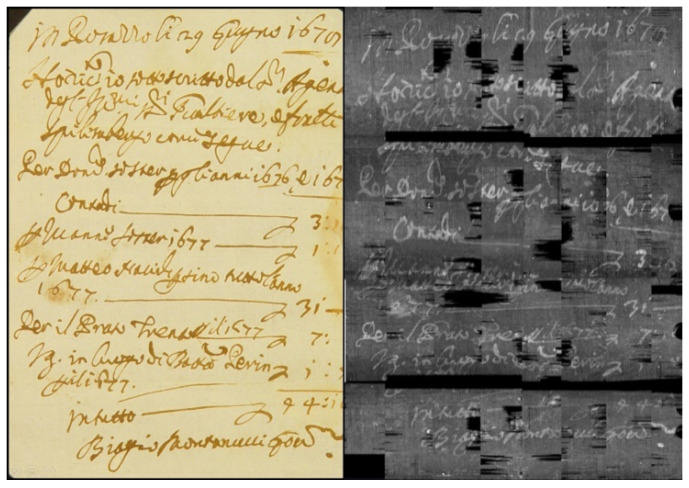
The result of the extraction of all the pages composing the document (**right**) compared with the unfolded original manuscript (**left**). The two black horizontal areas of larger size are due to the cropping of the slices in the pre-processing step, while the other black zones on the right are related to a failure of the propagation procedure in the area closest to the paper folds.

A detail of an extracted letter is shown in Figure 18. The texture of the paper is well-recovered, and the small light spots are due to metallic components of the ink and the paper impurities. In Figure 22, the result of the elaboration applied to the whole document is presented.

A quantitative evaluation of the wrongly extracted pixels has been performed, and has resulted in a correctness of reconstruction of around 93% of the whole document. The missing 7% is mostly localized close to the foldings. Compared with the state of the art (e.g., [25]), our algorithm provides better results, in terms of resolution and image definition: the grain of other texture is visible without any degradation thanks to the high signal-to-noise ratio; the ink distribution is not altered. Moreover, we underline that the techniques also works well when the distribution of the ink is not uniform on the page.

Moreover, the approach proposed in [34] is based on [40], in which convolution products are widely used for the calculation of the finite differences, a fact that increases the complexity of the algorithm and the execution time. Our method needs less than a couple of seconds to perform the extraction of a single layer, maintaining the time bounded execution.

**Figure 23:**
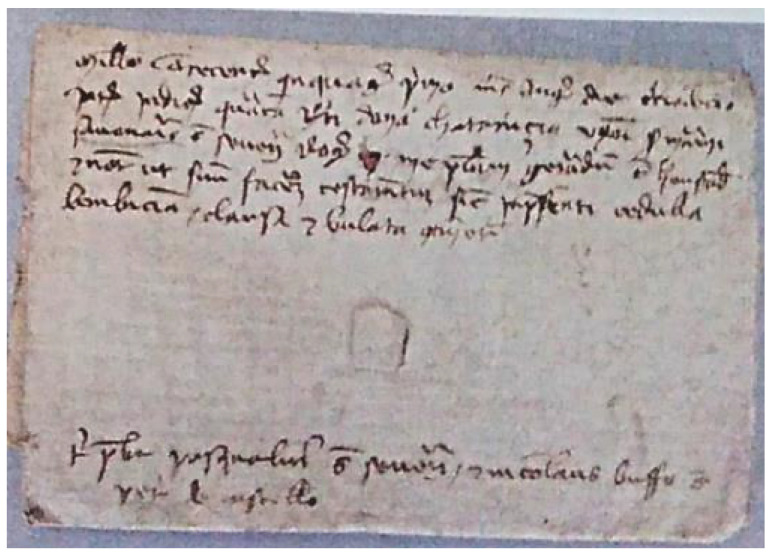
Envelope of the Venetian testament from the Archivio di Stato in Venice [29].

Finally, we provide the reconstruction and extraction of a sealed 1351 Venetian testament (original in Figure 23). The CT consists of 1534 slices, each one of 1500 × 200 pixels.

**Figure 24:**
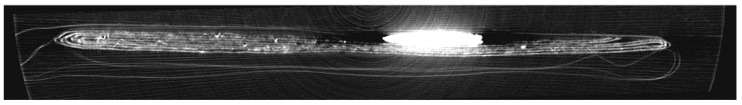
A single scan of the envelope of the Venetian testament from the Archivio di Stato in Venice. The image histogram has been stretched to put the envelope better in evidence. The seal (the big spot in white) and the ring artifacts are clearly visible.

In Figure 24, a single scan of the envelope is shown. It is evident how the general quality of the data are low due to the presence of ring artifacts and of spurious bright spots, probably due to the non-homogeneity of the used writing support. Due to the artifacts, to the noise and to the nature of the page the extraction appears challenging, if compared with other state-of-the-art cases (e.g., [34]).

**Figure 25:**
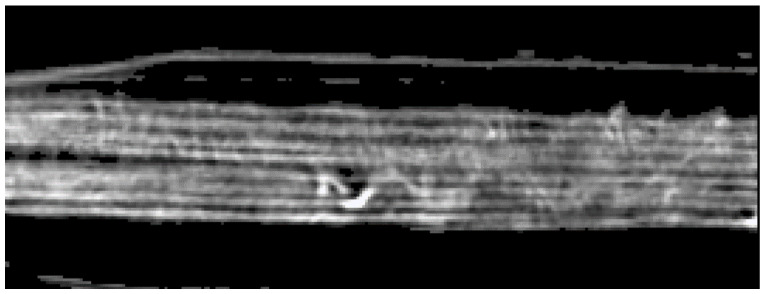
Sagittal section of the Venetian testament.

**Figure 26:**
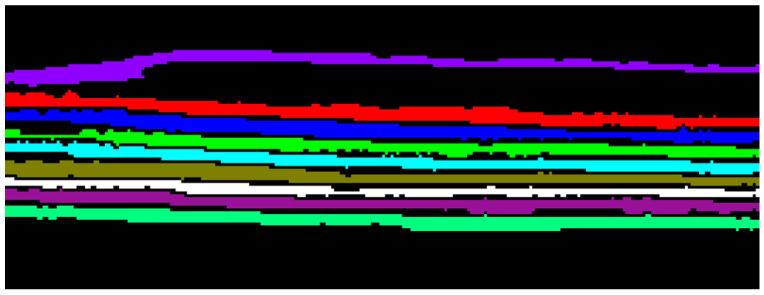
Segmentation result for the Venetian testament.

In Figure 25, a sagittal section of the reconstruction of the envelope is shown. Again, the non-homogeneity of the writing support poses a serious problem to the extraction of the pages. Subsequently, in Figure 26, the final segmentation of the pages is presented, each page with a different color.

**Figure 27:**
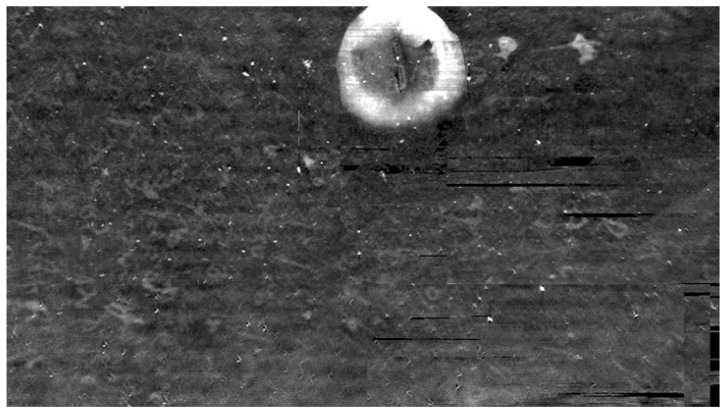
Reconstruction of one page, between two consecutive folds, of the Venetian testament.

**Figure 28:**
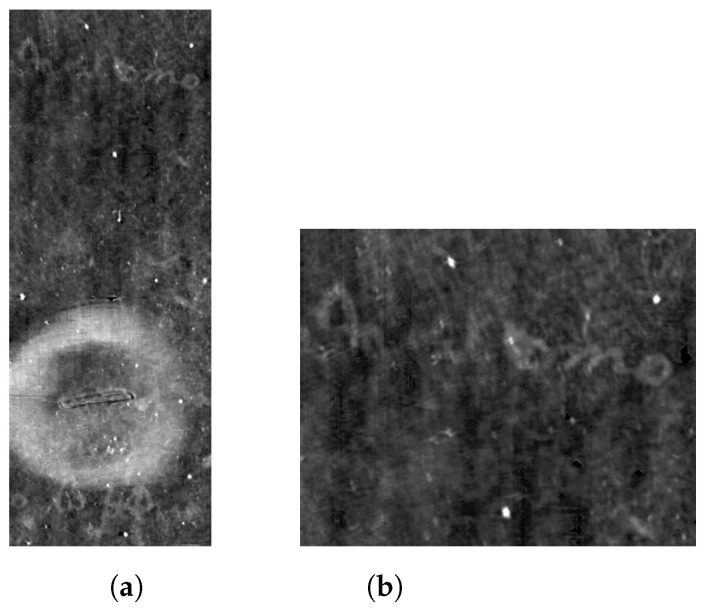
Details of the final extraction from Venetian testament. Even if the original scan is corrupted by artifacts and noise, the final result shows readable text. (**a**) Detail around the seal; (**b**) closer detail showing the text just over the seal.

In Figure 27 and Figure 28, the reconstruction, between two consecutive folds of the envelope, is shown.

The execution time for the extraction and the reconstruction of the Venetian testament is 3 min and 16 s for the whole document. This time can be further reduced parallelizing the software.

## 5. Conclusions

The methodology presented in this paper has been developed to recover text by means of CT acquisitions of ancient manuscripts. The quality of the recorded data, as well as the used image processing procedures, are equally important to perform a correct page extraction from the CT volumes. Improving some segmentation techniques, well known in the literature, we formulated a specific task-oriented algorithm that exhibits good performances in recognizing and separating the pages in the test document. To achieve the proposed goal, geometric definitions have been made on the morphology of the manuscript. Moreover, having used a single, specifically folded sheet, a further non-negligible problem is represented by the segmentation of the text in the folds of the pages. This issue is naturally absent in books, as their constructive morphology excludes the presence of meaningful text on the binding and the spine.

On the other hand, the lack of air between pages might introduce further difficulties connected with the less distinguishability among the pages themselves.

The achieved results show a good quality in the reconstruction, in terms of the main text and the texture.

The proposed method is semi-automatic because it leaves the possibility to choose the parameters to the user, which can not be a priori stated to be optimal, as in other state-of-the-art methods. Thanks to the fast execution time, different simulations can be operated, such that the user can choose the best combination of them case by case.

The execution time, compared with other state-of-the-art methods, is considerably lower, thanks to the simple computation of the introduced geometric features.

This study could be a starting point for scans of entire books; future implementations could consider the introduction of algorithms for inpainting of missing zones.

## Figures and Tables

**Table 1:** Tomographic settings of the document scan.

Voltage (kV)	Current (μA)	Projection (Number)	Range (°)	Voxel Size (μm)	Scan Time (h)
45	117	3000	360	15	9

**Table 2:** Geometric characteristics computed for each zone.

Zone	Area (Pixels)	Mean Depth Md (Pixels)	Standard Deviation
1	8004	3.3	1.3
2	4746	3.8	0.9
3	14,858	4.2	1.4
4	13,389	3.8	1.7
5	3836	2.8	0.4
Mean	8967	3.6	0.5

**Table 3:** Geometric features computed for each zone at the end of the separation step.

Zone	Area (Pixels)	Depth (Pixels)	Standard Deviation
1	3494	2.5	0.6
2	4428	3.4	0.8
3	4746	3.7	0.9
4	4374	3.4	0.8
5	4851	3.8	0.8
6	5527	4.5	0.8
7	4242	3.3	0.9
8	4553	3.5	0.8
9	4438	3.4	0.8
10	3836	2.8	0.4

## Data Availability

Not applicable.
